# Pediatric critical illness endotypes reveal distinct outcomes and immune pathways shared across cause of illness

**DOI:** 10.1016/j.isci.2025.114210

**Published:** 2025-11-26

**Authors:** Michael J. Carter, Joshua Hageman, Yael Feinstein, Jethro Herberg, Dominic Habgood-Coote, Victoria Wright, Samuel Nichols, Nazima Pathan, Naomi Edmonds, Philip D. Cowie, Katie Burnham, Alexander Mentzer, Julian Knight, Michael Levin, Myrsini Kaforou, Simon Nadel, Mark J. Peters, Padmanabhan Ramnarayan

**Affiliations:** 1 Department of Surgery and Cancer, Faculty of Medicine, Imperial College London, London, UK; 2 Paediatric Intensive Care Unit, Oxford University Hospitals NHS Foundation Trust, Oxford, UK; 3 Centre for Human Genetics, University of Oxford, Oxford, UK; 4 Department of Paediatrics, Gray Faculty of Medical and Health Sciences, Tel Aviv University, Tel Aviv-Yafo, Israel; 5 Section of Paediatric Infection, Faculty of Medicine, Imperial College London, London, UK; 6 Department of Paediatrics, University of Cambridge, Cambridge, UK; 7 Paediatric Intensive Care Unit, Royal London Hospital, Barts Health NHS Foundation Trust, London, UK; 8 Wellcome Sanger Institute, Wellcome Genome Campus, Hinxton, Cambridgeshire, UK; 9 Paediatric Intensive Care Unit, St Mary’s Hospital, Imperial College Healthcare NHS Trust, London, UK; 10 UCL Great Ormond Street Institute of Child Health, University College London, London, UK; 11 Children’s Acute Transport Service, Great Ormond Street Hospital, London, UK; 12 Centre for Paediatrics and Child Health, Imperial College London, London, UK

**Keywords:** Clinical genetics, Immunology, Pediatrics

## Abstract

Characterization of shared patterns of immune responses in critical illnesses, known as “endotypes,” may have therapeutic significance. Using unsupervised k-means clustering of genome-wide gene expression profiling, we derived, validated, and assigned endotype membership in 382 children with diverse critical illnesses recruited to the BASIC study. We identified two robust endotypes, BASIC endotype 1 (122, 31.9%, children) and BASIC endotype 2 (260, 68.1%, children), present in children with diverse illnesses and age groups. BASIC endotype 1 membership was associated with 4.1 days of increased duration of mechanical ventilation and a non-significant association with mortality. BASIC endotype 1 membership was associated with higher proportions of naive and resting memory CD4 T cells, lower proportions of neutrophils, and reduced expression of gene sets associated with tumor necrosis factor alpha (TNF-α), interferon-γ, interferon-α, and interleukin-6/JAK/STAT pathways in comparison with BASIC endotype 2. These BASIC endotypes may enable stratified trials of treatment for immune dysfunction.

## Highlights

• Features of immune dysregulation, “endotypes,” are shared in child critical illnesses
• Endotypes may be characterized by genome-wide gene expression profiling
• Endotype membership is associated with clinically important outcomes
• Gene set enrichment identifies target pathways for treatment of immune dysfunction

## Graphical abstract

**Figure:**
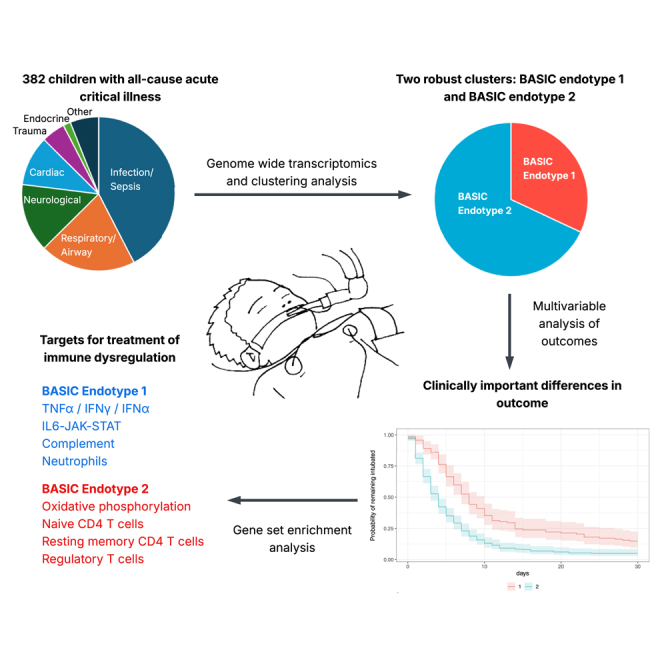

## Introduction

Acute critical illness is characterized by a need for life-sustaining organ support and may be caused by diverse infections, inflammation, trauma and surgery, and toxins.^1^ There is increasing recognition of shared immune responses across critical illnesses, including activation of the vascular endothelium, leukocyte activation and necrotic cell death, impaired antigen presentation, and dysfunctional lymphocytes.^2^^,^^3^^,^^4^ These shared patterns of immune responses were first termed endotypes following clustering of children with sepsis based on gene expression studies.^5^^,^^6^ Biological characterization of endotypes in adult and child sepsis,^7^^,^^8^^,^^9^^,^^10^ (pediatric) acute respiratory distress syndrome (ARDS),^11^^,^^12^ trauma,^13^ and burns^14^ at proteomic,^15^ cellular,^16^ and genomic levels^17^ has extended this concept to identify underlying mechanisms of disease. Several of these studies have sought to identify “treatable traits,”^1^ develop novel therapies, or repurpose existing drugs to treat dysfunctional immune responses in critical illnesses.^18^ However, few studies have sought to define shared immune responses across an inclusive population of children with all-cause critical illness.^1^

Extrapolation of data from studies of adult critical illness to childhood is problematic^3^ because of the rapid ontogeny of the immune system in infants and young children,^19^ leading to heterogeneity across age groups. Cohorts of children with critical illness may also be enriched for primary immunodeficiencies—including those not currently identified^20^—in comparison to adults. In addition, in children with community-acquired sepsis, more than half of deaths occur within 24 h of referral to critical care,^21^ while under one-fifth of adult deaths from community-acquired sepsis occur within 24 h.^22^ Yet sampling studies of gene expression in sepsis in both children and adults have been conducted after 24 h of referral, leading to a risk of survivor bias and potentially missing treatable traits early in the critical illness. Sampling for the development of endotypes using gene expression studies must therefore be age-appropriate, timely, identify shared biological mechanisms agnostic of specific cause of illness, and scalable to make rapid testing for stratification of patients into therapeutic trials feasible.^3^

We aimed to identify early, shared immune response features across diverse childhood critical illnesses. We undertook sampling of critically ill children during stabilization and retrieval to pediatric intensive care units (PICUs) for whole blood gene expression analysis. We developed and validated endotypes using unsupervised hierarchical clustering on these early whole blood gene expression data and analyzed endotype membership using detailed clinical data and outcome. We subsequently developed a continuous, quantitative score for endotype membership status, explored biological features of immune dysfunction characterized by the endotypes, and described their overlap with endotypes derived from studies of adults with sepsis.

## Results

### Recruitment and study cohort

During the study period, 1,017 eligible children were transported to participating PICUs via CATS, of whom 674 patients were recruited (Figure S1). Gene expression microarrays were performed on the first 382 patients, by recruitment date. Of the study cohort, 374 children (97.9%) were invasively ventilated at admission to PICU, and 222 (58.1%) required vasoactive support. The median duration of ventilation was 4 (interquartile range 2–8) days and the median ventilation-free days at day 30 (VFD-30) was 25 (interquartile range [IQR] 21–28) days, and 16 (4.2%) children died. Of the study cohort, 201 (52.6%) children were male, 205 (53.7%) children were <2 years of age, 75 (19.6%) were 2 years to <5 years of age, 78 (20.4%) were 5 years–12 years of age, and 24 (6.3%) were >12 years of age. The cause of critical illness was infection/sepsis in 162 (42.4%) children, respiratory/airway disease in 77 (20.2%), neurological in 55 (14.4%), and cardiac (including congenital cardiac disease) in 40 (10.5%), with smaller proportions of children with trauma/head injury, endocrine/metabolic disease, and other causes of critical illness (as per the UK PICAnet definitions^23^). Most children (296, 77.4%) had no comorbidity prior to admission. As determined by a standard algorithmic review of data following PICU outcome, 143 (37.4%) children had no infection, 91 (23.8%) children had viral infection, and 85 (22.2%) children had bacterial infection; the remaining 63 (16.5%) children had an indeterminate infection status. Full details of the BASIC cohort are shown in Table 1.

**Table 1:** Clinical characteristics of the study cohort

Clinical characteristic	All patients	BASIC endotype 1	BASIC endotype 2	*p* value
N	382	122 (31.9%)	260 (68.1%)	–
Sex at birth (%)				
Female	181 (47.4%)	54 (44.3%)	127 (48.8%)	0.467
Male	201 (52.6%)	68 (55.7%)	133 (51.2%)	–
Patient age group (%)				
0 to <1 month	78 (20.4%)	65 (53.5%)	13 (5.0%)	<0.001
1–11 months	74 (19.4%)	35 (28.7%)	39 (15.0%)	0.003
12–23 months	53 (13.9%)	3 (2.5%)	50 (19.2%)	<0.001
24 months–4 years	75 (19.6%)	8 (6.6%)	67 (25.8%)	<0.001
5 years–12 years	78 (20.4%)	7 (5.7%)	71 (27.3%)	<0.001
>12 years	24 (6.3%)	4 (3.3%)	20 (7.7%)	0.152
Reason for admission (%)				
Infection/sepsis	162 (42.4%)	41 (33.6%)	121 (46.5%)	0.023
Respiratory/airway	77 (20.2%)	27 (22.1%)	50 (19.2%)	0.602
Neurological	55 (14.4%)	7 (5.7%)	48 (18.5%)	0.002
Cardiac	40 (10.5%)	32 (26.2%)	8 (3.1%)	<0.001
Trauma/head injury	19 (5.0%)	1 (1.0%)	18 (6.9%)	0.021
Endocrine/metabolic	6 (1.6%)	1 (1.0%)	5 (1.9%)	0.713
Other	23 (6.0%)	13 (10.7%)	10 (3.8%)	0.697
Presence of 1 or more comorbidity (%)	193 (50.5%)	62 (50.8%)	131 (50.4%)	1
POPC status prior to admission				
No disability	296 (77.4%)	106 (86.9%)	190 (73.1%)	0.004
Mild disability	27 (7.1%)	8 (6.6%)	19 (7.3%)	0.958
Moderate disability	29 (7.6%)	3 (2.5%)	26 (10.0%)	0.017
Severe disability	30 (7.9%)	5 (4.1%)	25 (9.6%)	0.100
Infection status (%)				
Non-infectious	143 (37.4%)	60 (49.2%)	83 (31.9%)	0.002
Infection type unknown	63 (16.5%)	17 (13.9%)	46 (17.7%)	0.438
Viral infection	91 (23.8%)	26 (21.3%)	65 (25.0%)	0.509
Bacterial infection	85 (22.2%)	19 (15.6%)	66 (25.4%)	0.044
Pediatric ARDS status∗∗∗				
Missing pARDS status	77 (20.2%)	21 (17.2%)	56 (21.5%)	0.400
No pARDS	137 (35.9%)	40 (32.8%)	97 (32.7%)	0.457
Mild/moderate pARDS	123 (32.2%)	38 (31.1%)	85 (32.7%)	0.854
Severe pARDS	45 (11.8%)	23 (18.9%)	22 (8.5%)	0.006
PIM2 score (median, interquartile range [IQR])	0.057 (0.032–0.116)	0.080 (0.039–0.159)	0.052 (0.029–0.099)	<0.001
Investigations				
CRP at transfer (median, IQR, max)	28 (9–72, 520)	22 (9–52, 304)	36 (9–84, 520)	<0.001
Maximum CRP (median, IQR, max)	52 (20–134, 520)	47 (19–83, 304)	57 (21–143, 520)	<0.001
Neutrophil count at transfer (median, IQR, max)	7.2 (4.6–11.9, 50.8)	6.0 (3.2–10.3, 35.6)	7.6 (5.1–12.6, 50.8)	<0.001
Treatment				
Required invasive ventilation (%)	374 (97.9%)	119 (97.5%)	255 (98.1%)	1
Required vasoactive support (%)	222 (58.1%)	98 (80.3%)	124 (47.7%)	<0.001
Outcomes				
VFD-30 (days, median, IQR)	25.0 (21.0, 28.0)	22.0 (16.0–25.0)	26.0 (23.0–28.0)	<0.001
Duration of ventilation (days, median, IQR, max)	4.0 (2.0–8.0, 139)	6.0 (4.0–10.0, 139)	4.0 (2.0–7.0, 89)	<0.001
Mortality (%)	16 (4.2%)	8 (6.6%)	8 (3.1%)	0.190
Ventilation ≥30 days or mortality (%)	31 (8.1%)	18 (14.8%)	13 (5.0%)	0.002

All children on whom gene expression data were available and by BASIC endotype membership.

### Clinical characteristics of BASIC endotypes

**Figure 1:**
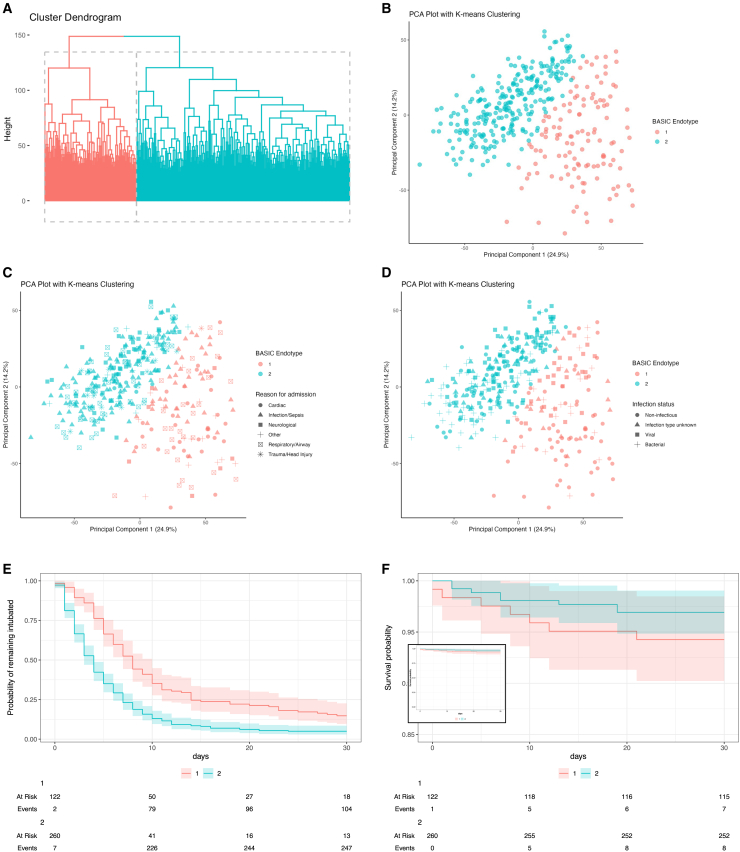
BASIC endotypes and outcomes (A) Hierarchical clustering figure of k-means nearest neighbor clustering (each sample shown as a single line on the dendrogram). (B) Principal-component analysis of k-means clustering to develop BASIC endotypes (each sample shown as a single point against principal components 1 and 2). (C) Principal-component analysis of k-means clustering with reason for admission to PICU. (D) K-means clustering with infection status. (E) Kaplan-Meier plot of extubation probability for BASIC endotype 1 (adjusted hazard ratio, HR, for extubation 0.47, 95% confidence intervals, CI, 0.14–1.60, p = 0.2; 95% CIs shown). (F) Kaplan-Meier plot of mortality for BASIC endotype 1 (HR 1.39, 95% CI 0.28–6.82, p = 0.7; 95% CIs shown).

We investigated heterogeneity in gene expression in the BASIC cohort. Initial analyses (Figures S2A and S2B) identified an optimal two clusters of patients. Patients were allocated to clusters, which we correspondingly termed BASIC endotype 1 (*n* = 122, 31.9%) and BASIC endotype 2 (*n* = 260, 68.1%), using k-means clustering (Figures 1A and 1B). BASIC endotypes (clusters) were validated using cross-validation (yielding accuracy 0.968) and a training (67%) and validation (33%) split (yielding accuracy of 0.976; Figures S2C–S2F).

BASIC endotype membership was closely associated with age of child, with a higher proportion of neonatal infants aged 0–1 month and infants aged 1–11 months in BASIC endotype 1 in comparison with BASIC endotype 2 (Table 1). There was a lower proportion of children in BASIC endotype 1 compared to BASIC endotype 2 among children diagnosed with infection/sepsis (41 [33.6%] versus 121 [64.5%], *p* = 0.023), neurological conditions (7 [5.7%] versus 48 [18.5%], *p* = 0.002), cardiac diagnoses (32 [26.5%] versus 8 [3.1%], *p* < 0.001), and trauma/head injury (1 [1.0%] versus 18 [6.9%], *p* = 0.021; Table 1). A higher proportion of children in BASIC endotype 1 had no pre-existing comorbidity in comparison with BASIC endotype 2 (106 [86.9%] versus 190 [73.1%], *p* = 0.004). A lower proportion of children in BASIC endotype 1 had a bacterial infection in comparison with BASIC endotype 2 (19 [15.6%] versus 66 [25.4%], *p* = 0.044; Figure 1D).

The pediatric index of mortality 2 (PIM2)^24^ score was higher for children in BASIC endotype 1 in comparison to BASIC endotype 2 (0.057, IQR 0.032–0.116, versus 0.080, IQR 0.039–0.159, *p* < 0.001). Of the 305 (79.8%) children on whom the oxygenation index (OI) or PaO_2_/FiO_2_ (P/F) ratio was calculated, the proportion of children with severe pediatric acute respiratory distress syndrome (pARDS)^25^ was higher in BASIC endotype 1 in comparison with BASIC endotype 2 (23, 18.9%, versus 22, 8.5%, *p* = 0.006). The median C-reactive protein (CRP) concentration at PICU admission was lower in children in BASIC endotype 1 compared with those in BASIC endotype 2 (22 mg/L, [IQR 9–52] versus 36 mg/L [IQR 9–84], *p* < 0.001) as was the median clinical laboratory neutrophil count (6.0 × 10^9^/L [IQR 3.2–10.3] versus 7.6 × 10^9^/L [IQR 5.1–12.6] , *p* < 0.001). Nearly all children in the cohort were invasively ventilated but a higher proportion of children in BASIC endotype 1 required vasoactive support in comparison with BASIC endotype 2 (98 [80.3%] versus 124 [47.7%], *p* < 0.001). The median PIM2 score was higher in children in BASIC endotype 1 in comparison with BASIC endotype 2 (0.08 [IQR 0.04–0.16] versus 0.05 [IQR 0.03–0.10], *p* < 0.001). Substantially similar comparisons between BASIC endotype 1 and BASIC endotype 2 members persisted when the analyses were limited to the 152 children with presumed infection at admission and PSS ≥2 (i.e., a diagnosis of sepsis,^26^; Table S1).

In a multiple variable regression model, adjusted for sex, age, reason for PICU admission, co-morbidity, infection status, and PIM2 score, the association of BASIC endotype 1 with younger age groups was confirmed (*p* < 0.001; Table 2). There was a positive association with cardiac reason for admission and BASIC endotype 1 (*p* = 0.011), with an association between youngest age group and cardiac reason for admission.

**Table 2:** Associations of demographic and clinical characteristics with BASIC endotype membership in multiple linear regression

Outcome	Variable	Estimate	95% CI	*p* value
BASIC endotype 1 membership	Sex			
	Female	–	–	–
	Male	−0.207	−0.85 to 0.42	0.522
	Age			
	0 to <1 month	–	–	–
	1–11 months	−1.581	−2.47 to 0.74	<0.001
	12–23 months	−4.557	−6.20 to 3.23	<0.001
	24 months–4 years	−4.104	−5.36 to 2.98	<0.001
	5–11 years	−4.254	−5.58 to 3.08	<0.001
	>12 years	−3.447	−5.12 to 2.01	<0.001
	Reason for PICU admission			
	Infection/sepsis	–	–	–
	Cardiac	1.736	0.31–3.26	0.020
	Endocrine/metabolic	0.934	−2.64 to 3.64	0.542
	Respiratory/airway	0.865	−0.07 to 1.81	0.069
	Neurological	0.009	−1.30 to 1.21	0.989
	Trauma/head injury	0.380	−2.75 to 2.60	0.763
	Other	1.326	−0.25 to 3.02	0.110
	Co-morbidity			
	None	–	–	–
	Cardiac	0.540	−0.83 to 2.07	0.456
	Endocrine/metabolic	0.426	−1.84 to 2.26	0.674
	Respiratory/airway	0.551	−0.50 to 1.59	0.300
	Neurological	−1.163	−3.20 to 0.41	0.188
	Hematology/oncology	2.896	1.17–4.66	0.001
	Genetic syndrome	0.631	−1.20 to 2.13	0.444
	Multi-system disorder	2.431	−0.80 to 5.05	0.074
	Other	1.349	0.42–2.33	0.005
	Infection status			
	Non-infectious	–	–	–
	Infection type unknown	0.498	−0.75 to 1.78	0.439
	Viral infection	0.486	−0.66 to 1.66	0.408
	Bacterial infection	0.776	−0.42 to 2.02	0.212
	PIM2 score (logit)	0.182	−0.04 to 0.40	0.105

### Outcomes by BASIC endotype membership

Median VFD-30 was 22 days (IQR 16–25) for children in BASIC endotype 1 in comparison to 26 days (IQR 23–28, *p* < 0.001) for children in BASIC endotype 2. In multiple variable regression, adjusted for age, reason for PICU admission, co-morbidity, infection status, and PIM2 score, VFD-30 was substantially lower in children in BASIC endotype 1 (estimate −3.7, 95% confidence interval [CI] −5.8 to −1.7, days, *p* < 0.001) in comparison with children in BASIC endotype 2 (Table 3). Results were substantially similar when adjusted for pARDS category or Phoenix Sepsis Score in relevant sub-cohorts of patients (Tables S2A and S2B). In an adjusted Cox proportional hazards model (with mortality treated as VFD-30 of 0), BASIC endotype 1 was associated with ventilation ≥30 days or mortality in comparison with BASIC endotype 2 (i.e., HR 1.50, 95% CI 1.10–2.06, *p* = 0.11; Figure 1E; Table 4).

**Table 3:** Ventilator-free days at day 30 by BASIC endotype membership

Outcome	Characteristic	Estimate	95% CI	*p* value
VFD-30	BASIC endotype 1 membership	−3.727	−5.757 to −1.697	<0.001
	Sex			
	Female	–	–	–
	Male	−1.665	−3.096 to −0.233	0.023
	Age			
	0 to <1 month	–	–	–
	1–11 months	1.095	−1.352 to 3.542	0.379
	12–23 months	2.077	−0.951 to 5.105	0.178
	24 months–4 years	−0.559	−3.399 to 2.282	0.699
	5 years–12 years	0.292	−2.596 to 3.181	0.842
	>12 years	0.787	−2.984 to 4.558	0.682
	Reason for PICU admission			
	Infection/sepsis	–	–	–
	Cardiac	−2.051	−5.426 to 1.325	0.233
	Endocrine/metabolic	2.464	−3.692 to 8.620	0.432
	Respiratory/airway	−0.792	−2.948 to 1.364	0.471
	Neurological	1.798	−0.806 to 4.402	0.175
	Trauma/head injury	0.950	−3.134 to 5.03	0.648
	Other	−1.165	−4.760 to 2.431	0.524
	Co-morbidity			
	None	–	–	–
	Cardiac	1.225	−2.383 to 4.833	0.505
	Metabolic/endocrine	−6.205	−10.53 to −1.878	0.00**5**
	Respiratory	−3.224	−5.661 to −0.787	0.010
	Neurological	−0.270	−3.006 to 2.467	0.846
	Hematology/oncology	−6.011	−11.13 to −0.89	0.022
	Genetic syndrome	−3.226	−6.596 to 0.144	0.061
	Multi-system disorder	3.210	−4.881 to 11.30	0.436
	Other	−1.140	−3.194 to 0.913	0.275
	Infection status			
	Non-infectious	–	–	–
	Infection type unknown	−0.686	−3.367 to 1.995	0.615
	Viral	−0.847	−3.297 to 1.603	0.497
	Bacterial	−1.288	−3.947 to 1.370	0.341
	PIM2 (logit)	−1.276	−1.821 to −0.73	<0.001

Multiple linear regression adjusted for age, reason for PICU admission, co-morbidity, infection status, and Pediatric Index of Mortality (PIM2) score (log-normalized).

**Table 4:** Outcome of PICU admission by BASIC endotype membership

Outcome	Characteristic	HR	95% CI	*p* value
	BASIC endotype 1 membership	1.39	0.28–6.82	0.7
(A) Mortality	Age			
	0 to <1 month	–	–	–
	1–11 months	0.59	0.09–3.67	0.6
	12–23 months	0.07	0–1.30	0.075
	24 months–4 years	0.39	0.06–2.60	0.3
	5 years–12 years	0.28	0.03–2.90	0.3
	>12 years	0	0–Inf	>0.9
	Reason for PICU admission			
	Infection/sepsis	–	–	–
	Cardiac	0.14	0.01–1.91	0.14
	Endocrine/metabolic	0	0–Inf	>0.9
	Respiratory/airway	0.75	0.11–5.34	0.8
	Neurological	0.38	0.03–4.88	0.5
	Trauma/head injury	0	0–Inf	>0.9
	Other	0.22	0.01–3.44	0.3
	Co-morbidity			
	None	–	–	–
	Cardiac	7.07	0.55–90.1	0.13
	Metabolic/endocrine	15.9	1.84–137	0.012
	Respiratory	6.13	0.90–41.8	0.064
	Neurological	3.98	0.33–47.7	0.3
	Hematology/oncology	0	0–Inf	>0.9
	Genetic syndrome	11.0	0.77–156	0.077
	Multi-system disorder	0	0–Inf	>0.9
	Other	7.77	1.33–45.3	0.023
	Infection status			
	Non-infectious	–	–	–
	Infection type unknown	0.35	0.04–3.31	0.4
	Viral	0.10	0.01–1.64	0.11
	Bacterial	0.58	0.08–4.22	0.6
	*PIM2 score (logit)*	2.20	1.60–3.03	<0.001
	*BASIC endotype 1 membership*	1.50	1.10–2.06	0.011
(B) Mortality or ventilation ≥30 days	Age			
	0 to <1 month	–	–	–
	1–11 months	1.27	0.87–1.86	0.2
	12–23 months	1.84	1.16–2.92	0.009
	24 months–4 years	1.34	0.87–2.07	0.2
	5 years–12 years	1.33	0.85–2.08	0.2
	>12 years	1.14	0.63–2.06	0.7
	Reason for PICU admission			
	Infection/sepsis	–	–	–
	Cardiac	0.67	0.41–1.09	0.1
	Endocrine/metabolic	0.85	0.34–2.12	0.7
	Respiratory/airway	0.78	0.56–1.10	0.2
	Neurological	1.67	1.15–2.43	0.007
	Trauma/head injury	1.1	0.59–2.04	0.8
	Other	0.55	0.31–0.96	0.035
	Co-morbidity			
	None	–	–	–
	Cardiac	1.01	0.60–1.72	>0.9
	Metabolic/endocrine	0.39	0.20–0.73	0.004
	Respiratory	0.75	0.52–1.07	0.11
	Neurological	1.15	0.75–1.75	0.5
	Hematology/oncology	0.48	0.22–1.05	0.068
	Genetic syndrome	0.57	0.34–0.95	0.031
	Multi-system disorder	2.07	0.61–6.99	0.2
	Other	0.75	0.55–1.02	0.068
	Infection status			
	Non-infectious	–	–	–
	Infection type unknown	0.63	0.42–0.97	0.03**5**
	Viral	0.59	0.41–0.86	0.006
	Bacterial	0.48	0.32–0.72	<0.001
	PIM2 score (logit)	0.86	0.79–0.93	<0.001

Cox proportional hazards model adjusted for age, reason for PICU admission, co-morbidity, or infection status and PIM2 score. (A) mortality as the event of interest (outcome). (B) Compound variable of mortality or ventilation ≥30 days as the event of interest (outcome).

Mortality was non-statistically significantly higher for children in BASIC endotype 1 in comparison with BASIC endotype 2 (8 [6.6%] versus 8 [3.1%], *p* = 0.190). In a Cox proportional hazard model (adjusted as above), there was no significant difference in mortality for children in BASIC endotype 1 in comparison to BASIC endotype 2 (hazard ratio [HR] 1.39, 95% CI 0.28–6.82, *p* = 0.7; Table 4).

### Quantitative analysis of BASIC endotypes

**Figure 2:**
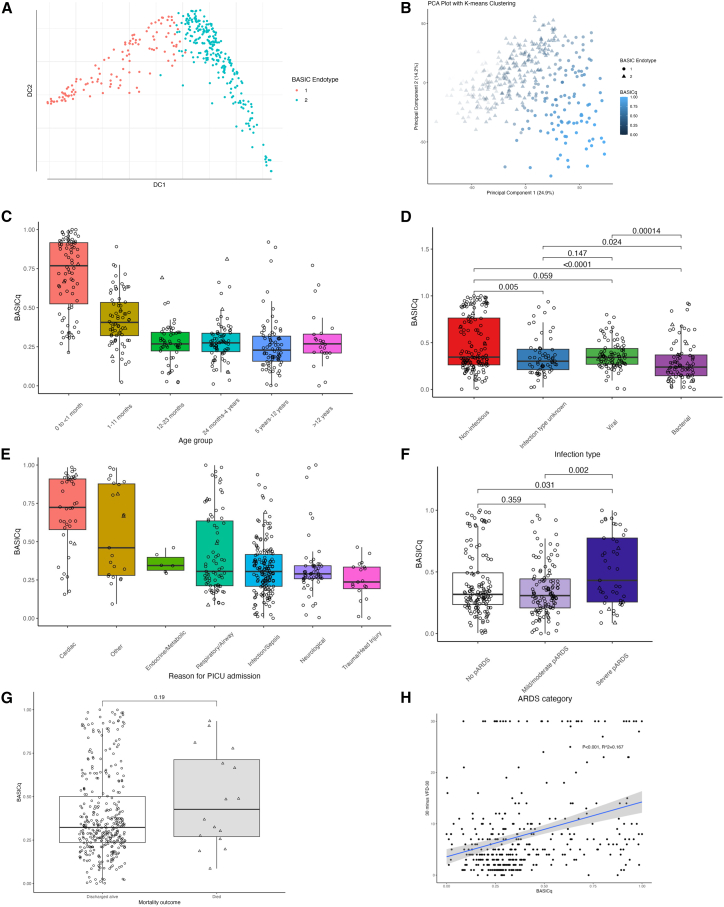
BASICq score and association with clinical features and outcomes (A) Use of diffusion mapping to develop a linear BASICq score. (B) Illustration of BASICq score by BASIC endotype on principal-component analysis plot. (C) BASICq score by age at admission to PICU. (D) BASICq score by cause of illness (*p* values from uncorrected Wilcoxon rank-sum tests). (E) BASICq score by reason for admission to PICU. (F) BASICq by pARDS status (*p* values from uncorrected Wilcoxon rank-sum tests). (G) BASICq score by mortality outcome (*p* value from uncorrected Wilcoxon rank-sum test). (H) BASICq score by length of invasive ventilation (censored at 30 days; linear regression line with 95% confidence intervals shown). For all boxplots, the hinge represents the median, the box represents the interquartile range (IQR), and the whiskers represent 1.5 times the IQR from the median.

We developed BASICq score as a quantitative continuous variable, normalized and limited between 0 and 1, as the first diffusion coefficient following diffusion mapping (Figures 2A and 2B).^8^^,^^27^ An increasing BASICq score indicated a trend toward BASIC endotype 1 membership. By age group, BASICq was highest in neonatal infants (0–1 month of age, median 0.77, IQR 0.52–0.92) in comparison with infants 1–11 months of age (median 0.41, IQR 0.32–0.53, *p* < 0.001), and infants in these two age groups had higher BASICq than all other age groups (which were similar by BASICq; Figure 2C; Table S3). BASICq was also higher in children with a non-infectious state (median 0.35, IQR 0.26–0.76) in comparison with unknown, viral or bacterial infection (Figure 2D; Table S4). By reason for admission to PICU, BASICq was highest in children with a cardiac reason for admission (median 0.72, IQR 0.58–0.91, and significantly higher than all other reasons for admission), followed by “other” reason for admission (median 0.46, IQR 0.28–0.56). All other reasons for admission had a similar BASICq (Figure 2E; Table S5). In children with OI or PaO_2_/FiO_2_ measured, BASICq was higher in children with severe pARDS than children with mild or moderate pARDS (*p* < 0.002) or no pARDS (*p* < 0.031; Figure 2F).

As a sensitivity analysis, we limited the analysis of BASICq to the 152 children with infection and PSS ≥2 (i.e., a diagnosis of sepsis; Table S1). In children with sepsis, BASICq was highest in neonatal infants (0 to <1 month of age, median 0.64, IQR 0.34–0.86) in comparison with infants 1–11 months of age (median 0.44, IQR 0.35–0.59, *p* < 0.030), and these infant age groups had higher BASICq than all other age groups (which were similar by BASICq; Figure S3; Table S6). BASICq score was similar across all levels of PSS (Figure S4; Table S7).

In multiple linear regression analyses, adjusted for age, reason for PICU admission, co-morbidity, and infection status, increased BASICq was associated with substantially lower VFD-30 (estimate −9.44, R^2^ 0.153, *p* < 0.001).

### Interpretation of BASIC endotypes

**Figure 3:**
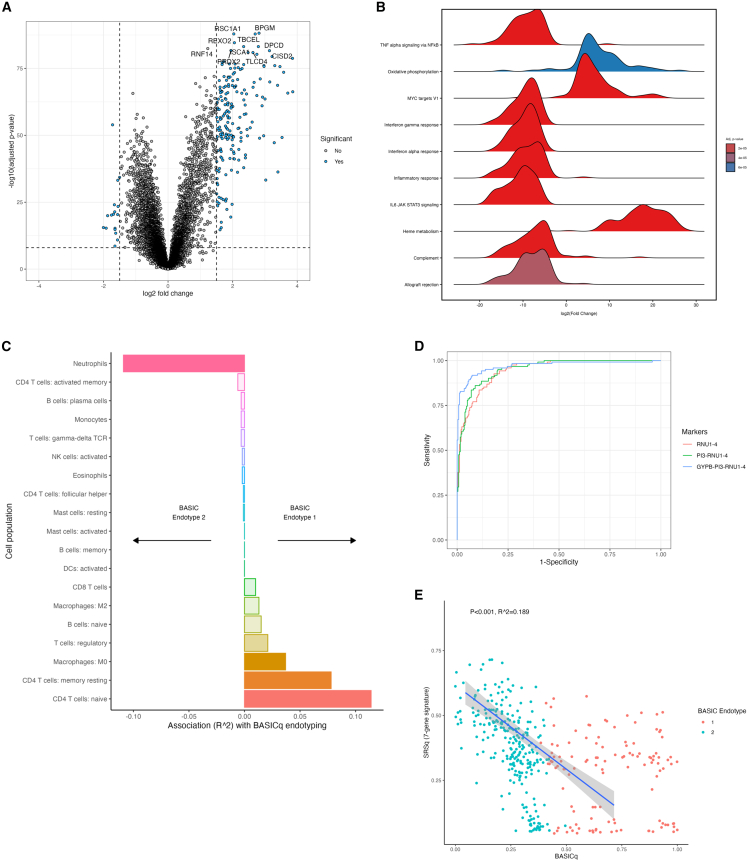
Biological interpretation of BASIC endotypes (A) Differential gene expression with upregulated genes in BASIC endotype 1 displayed to the right of the plot (Benjami-Hochberg adjusted *p* values; vertical lines at 1.5 log_2_ fold change, and horizontal line at adjusted *p* value 0.05). (B) Gene set enrichment analysis for top 10 differentially regulated gene sets by adjusted *p* value (ridge plot shows log_2_ fold change, with *p* value by color and distribution of genes within gene set plotted). (C) Association of imputed immune cell proportions (Cibersort) with BASICq score (as a quantitative measure of BASIC endotype membership, with *x* axis showing R^2^). (D) Receiver-operating characteristic (ROC) curves for optimal 1, 2, and 3 gene combinations selected by forward-selection-partial least squares (FS-PLS) to discriminate between BASIC endotypes. (E) Inverse association between BASICq derived from children with diverse critical illness and sepsis response score (SRS) 1 derived from adults with sepsis (linear regression with 95% confidence intervals shown).

Following removal of genes with zero expression status, and averaging of multiple probes to the same gene, all remaining 16,199 genes entered differential gene expression analysis and gene set enrichment analysis (GSEA).^28^ This identified many differentially expressed genes (log_2_ fold-change of 1.5 and Benjamini-Hochberg adjusted *p* value threshold of 0.05) in BASIC endotype 1 in comparison with BASIC endotype 2 (Figure 3A). The top 10 differentially enriched gene sets identified from GSEA included oxidate phosphorylation, targets of the transcription factor MYC, and heme metabolism (upregulated in BASIC endotype 1), as well as tumor necrosis factor alpha (TNF-α) via nuclear factor κB (NF-κB) signaling, interferon (IFN) γ and IFN-α response, inflammatory response, interleukin (IL)-6/JAK/STAT transcription factor, complement, and allograft rejection (all *p* < 0.005; Figure 3B).

We imputed the proportions of immune cell subsets using Cibersort.^29^ Imputed cell proportions were lower in correlation with the reference set (LM22^30^; i.e., less well-predicted) in BASIC endotype 1 in comparison with BASIC endotype 2. Imputed neutrophil proportions and imputed lymphocyte proportions were significantly correlated with neutrophil and lymphocyte proportions measured in clinical laboratory tests (Pearson’s r 0.558, *p* < 0.001 and Pearson’s r 0.672, *p* < 0.001, respectively; Figures S5A–S5C). When using BASICq as a continuous variable of endotype, neutrophils and activated CD4 T cells were all significantly associated with decreased BASICq (i.e., BASIC endotype 2 immune traits), and naive and memory resting CD4 T cells, M0 (naive) macrophages, and regulatory T cell proportions were all significantly associated with increased BASICq (i.e., BASIC endotype 1 immune traits; Figure 3C).

As a proof of concept, we developed a limited gene expression signature to discriminate between members of BASIC endotype 1 and 2. Of the 9,734 differentially expressed genes between BASIC endotype 1 and 2, we reduced the number of genes to the top 10 discriminatory genes (Figures S6A and S6B), before assessing the individual and combined diagnostic accuracy of a limited gene expression signature to discriminate between BASIC endotype 1 and 2. At an optimized (Youden) threshold, the two-gene expression signature *PI3–RNU1-4* had a sensitivity of 91.8% and specificity of 87.3%, and the three-gene expression signature *GYPB–PI3–RNU1-4* had a sensitivity of 92.6% and a specificity of 85.0% to discriminate between BASIC endotype 1 and 2 (Figures 3D, S6A, and S6B).

By applying a limited gene expression signature for adult sepsis response syndromes (SRS), we assessed the overlap of SRS to the BASIC dataset.^7^^,^^8^ We found overlap of the transcriptomic profile from children recruited to the BASIC cohort with SRS3 (healthy adult controls) and SRS2 (adults with sepsis and a relatively lower risk of mortality when admitted with sepsis) but not with SRS1 (adults with sepsis and a relatively high risk of mortality when admitted with sepsis; Figures S6C and S6D). We subsequently analyzed the association between BASICq (an increasing score that represents a higher likelihood of BASIC endotype 1 membership, which in turn is associated with increased length of ventilation) and SRSq (an increasing score that represents increased likelihood of SRS1 membership). There was an inverse association between BASICq and SRS1 (R^2^ 0.18, *p* < 0.001; Figure 3E).

## Discussion

We aimed to identify early shared immune response features across diverse childhood critical illnesses using whole blood gene expression analyses from 382 children admitted to PICU following emergency transport.^31^ These shared immune responses have been termed endotypes,^5^^,^^6^ and their existence across diverse critical illnesses has been hypothesized but not previously confirmed.^1^ The characterization of these immune features is important to explore the “treatability” of immune dysfunction across diverse clinical syndromes.

Here, we developed binary BASIC endotypes using k-means clustering that were robust to a training-validation split and cross-validation. Membership of BASIC endotype 1 was associated with fewer VFD-30 (i.e., increased length of ventilation by ∼4 days, with patient mortality treated as VFD-30 of 0). There was no significant association with a mortality outcome in this cohort, with a baseline mortality of 4.2%. BASIC endotype membership was associated with age of child at sampling, with 82% of children in BASIC endotype 1 aged <1 year. BASIC endotype membership likely partly represents immune maturation. However, the association of BASIC endotype 1 membership with fewer VFD-30 was robust to reason for admission, co-morbidity status, infection status (including etiology of infection), and conventional markers of childhood critical illness severity, such as PIM2 score.^24^^,^^26^^,^^32^ In a subset of 152 patients with sepsis (using PSS criteria^26^), BASIC endotype membership (and BASICq) was associated with age, but not increasing PSS, suggesting that BASIC endotypes represent an element of disease severity that is not represented by the PSS. Our initial biological exploration of the BASIC endotypes suggested that BASIC endotype 2 was associated with increased immune cell activation, including enrichment of gene sets associated with inflammation and elevated proportions of neutrophils and activated CD4 memory T cells. This contrasts with BASIC endotype 1, which was associated with decreased immune cell activation and elevated proportions of naive and resting memory CD4 T cells, and M0 macrophages. BASIC endotypes were developed on genome-wide gene expression data, but the high collinearity between gene expression makes a limited gene set—with potentially clinically relevant turnaround times—feasible. Here, we show a three-gene set signature that discriminates between endotypes with ∼93% sensitivity and 85% specificity to illustrate the potential of this approach for future stratified clinical trials.

Characterization of endotypes by etiology, severity, and treatability of disease may be important to identify treatable immune dysfunction in childhood critical illness.^3^^,^^33^^,^^34^ However, with exceptions,^6^^,^^10^^,^^35^^,^^36^^,^^37^ data have been developed in adults and almost entirely in sepsis.^7^^,^^8^^,^^9^^,^^38^ This limits the applicability of adult-derived data to childhood critical illness, and limitation to sepsis hampers the identification of shared immune dysfunction across critical illnesses. In addition, most child and adult cohorts have also been sampled typically 1–2 days following admission to intensive care unit (ICU) with a consequential risk of survivor bias, particularly in children, where a greater proportion of mortality occurs within the first 24 h of (P)ICU admission in comparison to adults.^21^^,^^22^ Intriguingly, (age-associated) BASIC endotypes were inversely associated with SRS status in adults, suggesting age-associated mechanisms to disease severity in patients with sepsis. In contrast, our disease agnostic and early sampling protocols have enabled exploration of early gene expression changes and the development of endotypes of diverse childhood critical illnesses.

A key question is whether endotypes can be used to predict a response to treatment. A *post hoc* analysis of a randomized controlled trial of vasopressin versus norepinephrine and hydrocortisone versus placebo (the VANISH trial) identified increased mortality in adults with sepsis in SRS2 endotype in response to hydrocortisone administration in comparison to placebo. There was no increased mortality in response to hydrocortisone administration in SRS1 endotype members.^39^ This was interpreted as harm from hydrocortisone administration in adult septic patients with less immune dysfunction (SRS2) in comparison to patients with an immunosuppressed endotype (SRS1). In contrast, the application of pediatric-sepsis-derived endotypes (endotype A, characterized by increased risk of mortality and suppression of adaptive immunity and endotypes B and C with less immune dysfunction)^5^ to the VANISH trial data showed the converse: odds of mortality were three times greater in response to hydrocortisone administration in members of endotype A (suppressed adaptive immunity) in comparison with members of endotype B and C.^40^ These data suggest that treatable traits do exist in sepsis, and, more specifically, adult-sepsis-derived endotypes encompass immune features that have a different response to hydrocortisone in comparison to pediatric-sepsis-derived immune features. Here, we show evidence that shared immune features are applicable across childhood critical illness, inferring that these treatable traits may also be more broadly applicable. Endotyping may therefore be directly applicable to future (platform) trials in childhood critical illness.

### Limitations of the study

Our study is illustrative of an approach to endotyping in diverse childhood critical illnesses, but there are several limitations. Our study is based on a single cohort of patients—although patients were subsequently transferred to one of four PICUs—and refinement and validation of early endotypes across critical illnesses in future cohorts is needed. RNA sequencing technologies have largely replaced genome-wide microarray techniques. We are therefore translating BASIC endotyping methods to RNA sequencing data to enable further external validation on large cohorts of critically ill children and adults.^8^ Despite the early sampling and deferred consent, consent was declined for 114 children, and 30 children were ineligible for analysis due to their death before consent was obtained from parents. Despite our best efforts, there may still be a residual survivor bias in the cohort, and not unexpectedly, the study is underpowered to detect a significant difference in mortality between endotypes. Although we have minimized the risk of treatment effect (by sampling patients early in disease), exploring the immune trajectory of childhood critical illness is a specific goal in future work to understand both effects of endotype membership and of immunomodulatory treatment in childhood critical illness in detail. Repeated measurements on the same patient will also enhance the statistical power to assess treatment effects and should be incorporated into future trials. The BASIC study also used only a single “omic” technology: the addition of single-cell analyses,^4^ and/or proteomics,^41^ and their integration^1^^,^^3^^,^^42^ would yield further insight that may be important in refining “treatability.” In particular, the role of neutrophils—and their interactions with other immune cells—has only recently started to be explored,^43^ and yet neutrophil proportions and activation made the strongest contribution to BASIC endotype 2 membership status in our study and conversely made the greatest contribution to SRS1 in adult sepsis.

In conclusion, we have shown endotypes that are applicable across diverse etiologies of childhood critical illness, which are associated with the clinically relevant outcome of VFD-30 and have basis in shared immune features. The development, refinement, and use of endotypes in observational research and in stratified clinical trial platforms may accelerate research in childhood critical care by increasing the signal of treatment effects and potentiating more effective clinical care in future.

## Resource availability

### Lead contact

Further information and requests for resources and reagents should be directed to and will be fulfilled by the lead contact, Dr Michael Carter (michael.carter@well.ox.ac.uk).

### Materials availability

This study did not generate new unique reagents.

### Data and code availability

• Data: gene expression data have been deposited at Annotare (https://www.ebi.ac.uk/fg/annotare/) with the accession code of Annotare: E-MTAB-15164 and are publicly available as of the date of publication. The clinical data reported in this study cannot be deposited in a public repository because of patient confidentiality. Clinical data reported in this paper will be shared by the lead contact upon request.
• Code: all original code has been deposited at https://github.com/michaeljamescarter/BASIC and is publicly available at https://doi.org/10.5281/zenodo.17438307 as of the date of publication. Details are also in the key resources table.
• Other: please see the key resources table for details of other resources and reagents used.

## Acknowledgments

We thank all the parents and children participating in this study and the medical, nursing, and research teams at the participating sites for their help in study setup, recruitment, data collection, and monitoring of study data for their invaluable support. M.J.C., A.M., and J.K. receive funding via the 10.13039/100024063National Health Service Genomic Network of Excellence Award for Severe Presentations of Infectious and Inflammatory Disease. J.Hageman., J.Herberg., and P.R. received support from the Great 10.13039/501100008174Ormond Street Hospital Children’s Charity. P.R., J.Herberg., M.L. and M.K., and M.J.P. receive funding from the 10.13039/501100000272National Institute for Health and Care Research (NIHR), in part through the Imperial-NIHR (P.R., J.Herberg., M.L., M.K.) and UCL-NIHR 10.13039/100014461Biomedical Research Centres (M.J.P.), respectively. K.B. is a member of the Wellcome Sanger Institute, which is funded by the 10.13039/100010269Wellcome Trust (220540/Z/20/A). For Open Access, the authors have applied a CC BY public copyright license to any Author Accepted Manuscript arising from this submission.

## Author contributions

M.J.C., J.Hageman., K.B., J.K., M.L., J.Herberg., M.K., and P.R. contributed to the conceptualization and design of the study. M.J.C., J.Hageman., D.H.C., K.B., M.K., and P.R. were responsible for acquisition, analysis, or interpretation of data. Y.F., J.Herberg., V.W., S.N., N.P., N.E., M.L., S.N., M.J.P., and P.R. were responsible for clinical investigation. M.J.C., J.Hageman., and K.B. were responsible for visualization of data. P.R., M.L., J.Herberg., M.K., and M.J.P. were responsible for funding acquisition. P.R., M.J.C., M.K., K.B., and M.L. were responsible for supervision. Writing of the original draft was led by M.J.C., J.Hageman., K.B., and P.R. Review and editing of subsequent drafts was led by M.J.C., J.Hageman., Y.F., J.Herberg., D.H.C., V.W., S.N., N.P., N.E., K.B., A.M., J.K., M.L., M.K., S.N., M.J.P., and P.R. M.J.C., J.H., M.K., and K.B. have directly accessed and verified the underlying data in the manuscript. All authors have had full access to the data.

## Declaration of interests

We declare no competing interests.

## STAR★Methods

### Key resources table

**Table:** 

REAGENT or RESOURCE	SOURCE	IDENTIFIER
**Biological samples**		

Whole blood from participants	Prospective recruitment for the BASIC study. Great Ormond Street Hospital for Children NHS Foundation Trust	Biomarkers of Acute Serious Illness in Children (BASIC). NCT03238040

**Critical commercial assays**		

PAXgene Blood RNA Tubes	Qiagen	Cat#762165
PAXgene Blood miRNA Kit	Qiagen/ PreAnalytiX	Cat#763134
HumanHT-12 v4 BeadChip	Illumina	https://www.illumina.com/documents/products/product_information_sheets/product_info_humanht-12.pdf

**Deposited data**		

Normalized gene expression data included in the analyses	https://www.ebi.ac.uk/fg/annotare/	Accession code: E-MTAB-15164

**Software and algorithms**		

R statistical program package	R Core Team (2024)^44^	https://www.r-project.org/
CIBERSORTx	CIBERSORTx. Stanford University^45^	https://cibersortx.stanford.edu/
Lumi v1.1.0	Du et al.^46^	https://www.bioconductor.org/packages/release/bioc/html/lumi
Parsnip v1.1.2	Kuhn and Wickham^47^	https://github.com/tidymodels/parsnip/releases
Cluster profiler v4.12.3	Yu et al.^48^	https://bioconductor.org/packages/clusterProfiler/
Human MSigDB Collections	Subramanian et al.^28^	https://www.gsea-msigdb.org/gsea/msigdb/collections.jsp
SepstratifieR	Cano-Gamez et al.^8^	https://github.com/jknightlab/SepstratifieR.git
CombiROC v0.3.4	Mazzara et al.^49^	http://combiroc.eu/
Limma v3.60.3	Ritchie et al.^50^	https://bioconductor.org/packages/limma/

**Other**		

Code used for the analyses described in the manuscript	N/A	All original code has been deposited at https://github.com/michaeljamescarter/BASIC and is publicly available at https://doi.org/10.5281/zenodo.17438307 as of the date of publication.

### Experimental model and study participant details

Demographic data and clinical characteristics of all study participants is presented in the main text Table 1 and the study recruitment flow chart is presented in Figure S1. We analysed data collected as part of the Biomarkers of Acute Serious Illness in Children (BASIC) study.^31^ BASIC was a prospective cohort study that enrolled critically ill children admitted to four PICUs in London and Eastern England, UK, during emergency transport by the Children’s Acute Transport Service (CATS), the regional paediatric critical care retrieval service (Figure S1) from 2014–2016. Children aged 0–16 years with an indwelling central venous or arterial catheter were eligible. Infants <36 weeks of corrected gestational age and those with agreed do-not-attempt-resuscitation plans were not recruited. The study was approved by the Health Research Authority (HRA, reference: 136866) and ethical approval was provided by the National Research Ethics Committee East Midlands—Nottingham 2 (reference: 13-EM-0399). Recruitment followed deferred consent processes, with written informed consent obtained by the study research team from parents/legal guardians within 24–48 hours.^44^ The study reporting follows the Standards of Reporting of Diagnostic Accuracy Studies 2015 Update (https://www.equator-network.org/reporting-guidelines/stard/).

### Method details

#### Data

We used patient demographic data (sex at birth, age, self-reported ethnicity), clinical characteristics (reason for PICU admission, duration of acute illness prior to transport, pre-morbid Pediatric Overall Performance Category), details of infection status (including phenotyping for bacterial, viral, unknown and non-infectious illnesses using standardized published criteria^45^), clinical severity markers such as the Paediatric Index of Mortality (PIM-2)^24^ score, retrospectively applied Phoenix Sepsis Score (PSS)^26^ and paediatric acute respiratory distress syndrome (pARDS),^32^ at admission to PICU as appropriate, interventions performed (invasive ventilation, vasoactive drugs) during transport and PICU discharge outcome (vital status and duration of organ support).

#### Microarrays

Whole blood (2.5 ml) was sampled into RNA-stabilising tubes (PAXgene, Qiagen, Germany) from recruited patients immediately during paediatric critical care retrieval. Samples were initially stored on ice packs (4°C) prior to arrival to the admitting PICU, before storage at -80°C and processing in batches. We purified total RNA using PAXgene Blood miRNA kits (PreAnalytix) as outlined in the manufacturer protocol and used Illumina Human-HT-12 version 4 Expression BeadChips with 47231 probes (Illumina San Diego, CA, USA) for genome-wide gene expression profiling of samples taken during retrieval following the protocol as outlined by the manufacturer.

### Quantification and statistical analysis

Statistical analysis was undertaken in R: A Language and Environment for Statistical Computing (version 4.4.1),^46^ with the exception of Cibersort (https://cibersortx.stanford.edu/).^29^ Microarray data were compiled, log_2_ transformed and normalised with robust spline normalization with *lumi* (version 1.1.0).^47^ Transcript probes that had non-zero expression status were taken forward for the development of BASIC endotypes, and for the assessment of adult-derived sepsis response syndrome (SRS) endotypes in the BASIC cohort. The mean expression of probes that matched to the same gene was used for all analyses.

We used unsupervised K-means clustering of gene expression microarray data for the development of the BASIC endotypes. Following previous work,^7^^,^^9^ we used the top 15% (∼5000) most variable transcript probes in the dataset and used sum of squares, minimum silhouette and the Gap statistic to optimise cluster numbers before using k-means clustering (Hartigan and Wong algorithm) with two centres to determine BASIC endotype membership for individual children at retrieval to PICU. BASIC endotype membership was internally validated with a train/validation (2/3 training, 1/3 validation) partition of the dataset, and a nested cross-validation implementation of GLMNet, *nestedcv*^48^ (*parsnip*, version 1.2.1, Figures S2A–S2F).

Comparisons of clinical data between BASIC endotypes were prepared using non-parametric (Wilcoxon Rank Sum and Kruskal-Wallis) methods. We used ventilation-free days at day 30 as the primary outcome (VFD-30, a measure of duration of ventilation used in cohorts with a relatively low incidence of mortality). VFD-30 was used instead of PICU-free days at day 30 since VFD-30 is less dependent upon step-down capacity in regional hospitals. Mortality (within 30 days), and mortality or ventilation ≥30 days were used as secondary outcomes. We undertook a sensitivity analysis of membership of BASIC endotypes in patients with sepsis,^26^ and pARDS.^32^

We explored BASIC endotype membership as a continuous quantitative variable using diffusion mapping used the R package *destiny*^27^ to implement diffusion mapping^8^; we termed this variable BASICq. To explore the biological differences between BASIC endotypes, we implemented differential gene expression analysis of all genes with non-zero expression status with *limma*^28^ (version 3.60.3) and gene set enrichment analysis (GSEA,^28^ as implemented in *clusterProfiler*,^8^ version 4.12.3) using the C7 immunologic signature gene set available from the Human MSigDB Collections (https://www.gsea-msigdb.org/gsea/msigdb/collections.jsp). Following (mean) averaging of probes that mapped to the same gene and removal of non-mapped probes, all probes with non-zero expression status were used for differential gene expression analysis. We also used Cibersort (https://cibersortx.stanford.edu/),^29^ using the reference immune cell set LM22,^30^ to infer differences in immune cell abundance between BASIC endotypes. To develop the limited gene expression signature, we used forward selection partial least squares (FS-PLS) as implemented by the R package *mt*^49^ (version 2.0-1.2), and *combiroc*^50^ (version 0.3.4) to determine the optimum combination of markers. We subsequently implemented the R package *SepstratifieR*^8^ to assess the overlap of BASIC endotype membership with SRS endotype membership in children in the BASIC study (with and without a diagnosis of sepsis).

### Additional resources

Clinical trials registry for Biomarkers of Acute Serious Illness in Children (BASIC) study: https://clinicaltrials.gov/study/NCT03238040, trial number: NCT03238040. Data presented in this paper represents a retrospective analysis of data collected during the BASIC study.
