# Supplementary material for: Pediatric critical illness endotypes reveal distinct outcomes and immune pathways shared across cause of illness
Source: iScience. 2025 Nov 26;28(12):114210. doi: 10.1016/j.isci.2025.114210 (PMC12756572; doi:10.1016/j.isci.2025.114210)
Supplement: Document S1. Figures S1–S6 and Tables S1–S7 [file mmc1.pdf]

iScience, Volume *28*

Supplemental information

Pediatric critical illness endotypes reveal distinct outcomes and immune pathways shared across cause of illness

Michael J. Carter, Joshua Hageman, Yael Feinstein, Jethro Herberg, Dominic Habgood-Coote, Victoria Wright, Samuel Nichols, Nazima Pathan, Naomi Edmonds, Philip D. Cowie, Katie Burnham, Alexander Mentzer, Julian Knight, Michael Levin, Myrsini Kaforou, Simon Nadel, Mark J. Peters, and Padmanabhan Ramnarayan

SUPPLEMENTAL INFORMATION

Supplemental Figures

Supplemental Figure 1. Recruitment of patients to the cohort.

Supplemental Figure 2. Development of clusters, and their cross-validation.

Supplemental Figure 3. BASICq score by age group for children meeting a diagnosis of sepsis at admission to PICU.

Supplemental Figure 4. BASICq score by Phoenix Sepsis Score (PSS).

Supplemental Figure 5. Use of Cibersort to impute immune cell proportions in data.

Supplemental Figure 6. Development of a limited gene signature set and comparison with sepsis response syndromes in adults with sepsis.

Supplemental Tables

Supplemental Table 1. Children on whom infection was suspected or confirmed and with a Phoenix Sepsis Score ≥2 (established sepsis).

Supplemental Table 2A. Ventilator-free days at day 30 (VFD-30) by BASIC endotype membership.

Supplemental Table 2B. Ventilator-free days at day 30 (VFD-30) by BASIC endotype membership.

Supplemental Table 3. Comparison of BASICq score by age group.

Supplemental Table 4. Comparison of BASICq score by infection status.

Supplemental Table 5. Comparison of BASICq score by reason for admission.

Supplemental Table 6. Comparison of BASICq score by age group in patients with infection and PSS ≥2 (i.e. a diagnosis of sepsis).

Supplemental Table 7. Comparison of BASICq score by Phoenix Sepsis Score (PSS) in patients with infection and PSS ≥2 (i.e. a diagnosis of sepsis).

SUPPLEMENTAL FIGURES

Supplemental Figure 1. Recruitment of patients to the cohort.

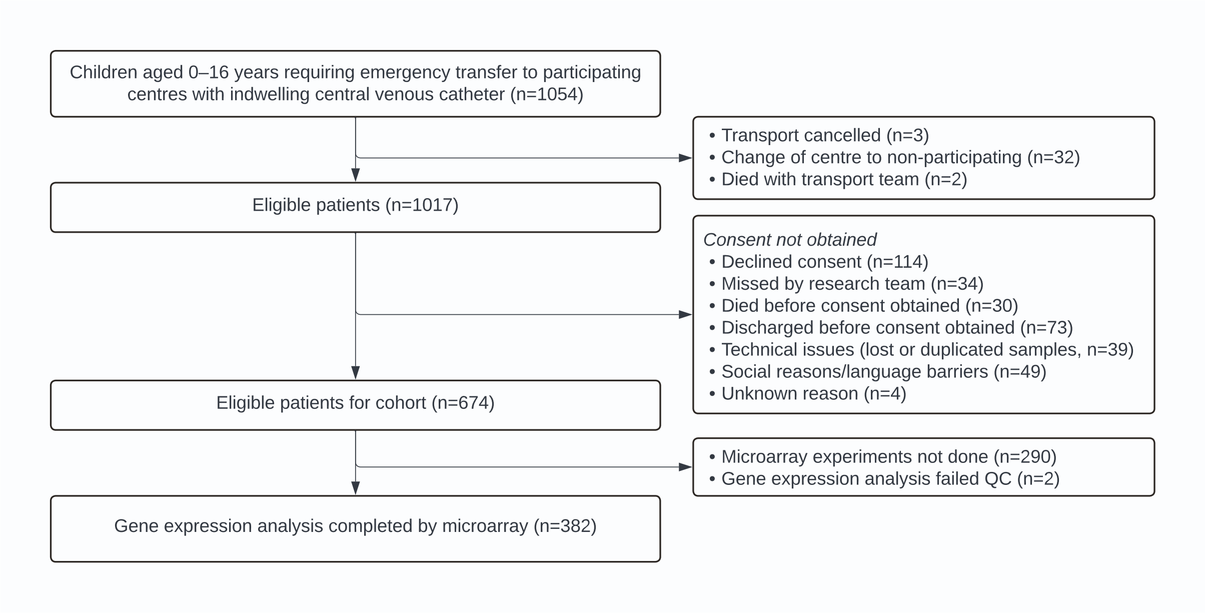

Supplemental Figure 2. Development of clusters, and their cross-validation. (A) Scree plot of percentage of variance explained by dimensions 1–10. (B) Average silhouette width for optimum cluster number. (C) and (D) binomial deviance as a marker of model fit for varied α and λ values in *GLMnet*. (E) receiver-operating characteristic curve for predicting BASIC endotype using full RNA expression signatures. (F) Cross validation of the full RNA expression signature for BASIC endotype status with *GLMnet* and with a random training (67%) and validation (33%) split.

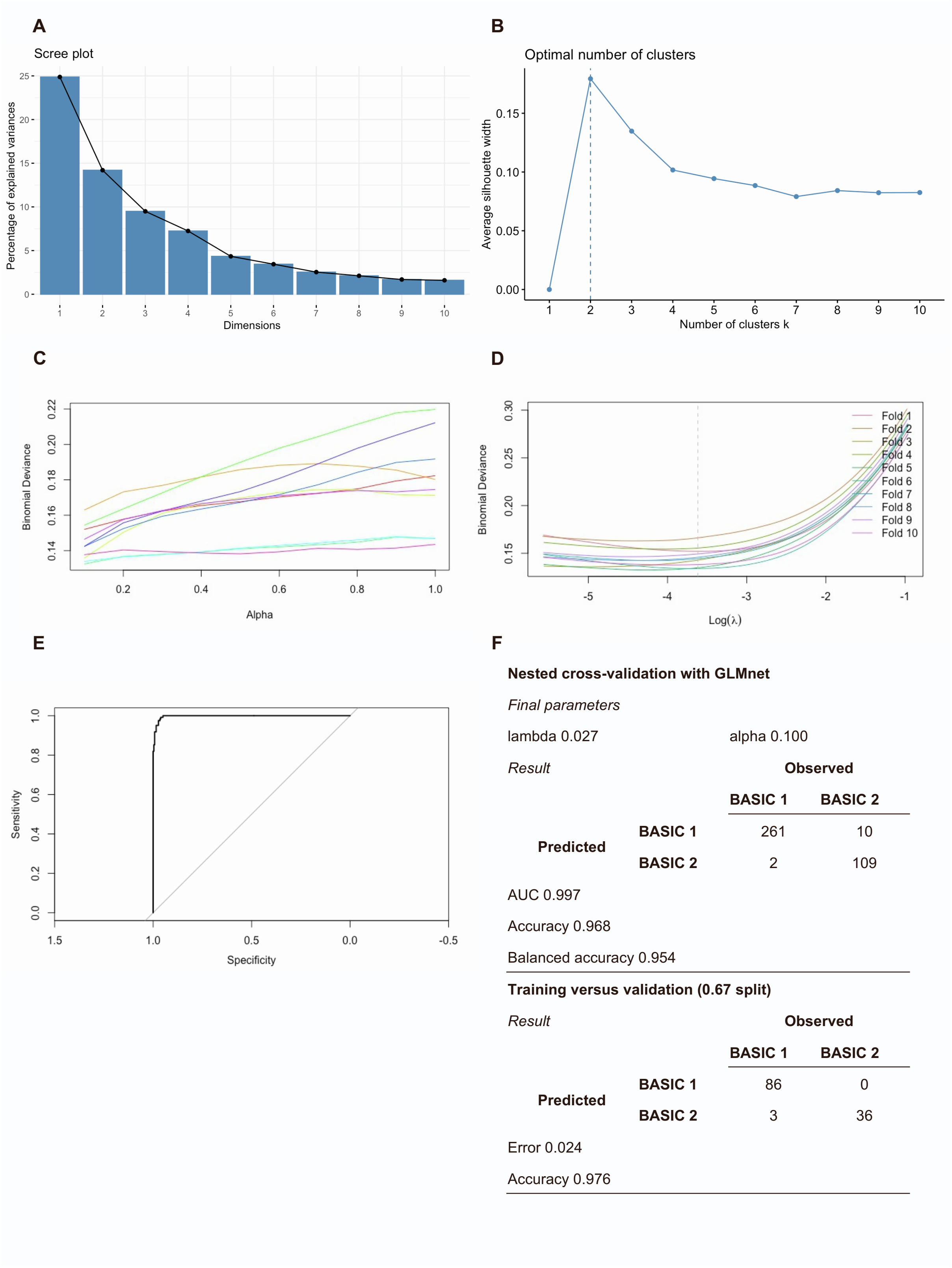
A
Scree plot
Percentage of explained variances
Dimensions
B
Optimal number of clusters
Average silhouette width
Number of clusters k
C
Binomial Deviance
Alpha
D
Binomial Deviance
Log(λ)
Fold 1
Fold 2
Fold 3
Fold 4
Fold 5
Fold 6
Fold 7
Fold 8
Fold 9
Fold 10
E
Sensitivity
Specificity
F
Nested cross-validation with GLMnet
Final parameters
lambda 0.027
alpha 0.100
Result
Observed
BASIC 1
BASIC 2
Predicted
BASIC 1
261
10
BASIC 2
2
109
AUC 0.997
Accuracy 0.968
Balanced accuracy 0.954
Training versus validation (0.67 split)
Result
Observed
BASIC 1
BASIC 2
Predicted
BASIC 1
86
0
BASIC 2
3
36
Error 0.024
Accuracy 0.976

Supplemental Figure 3. BASICq score by age group for children meeting a diagnosis of sepsis at admission to PICU. Patients with infection and PSS ≥2 only.

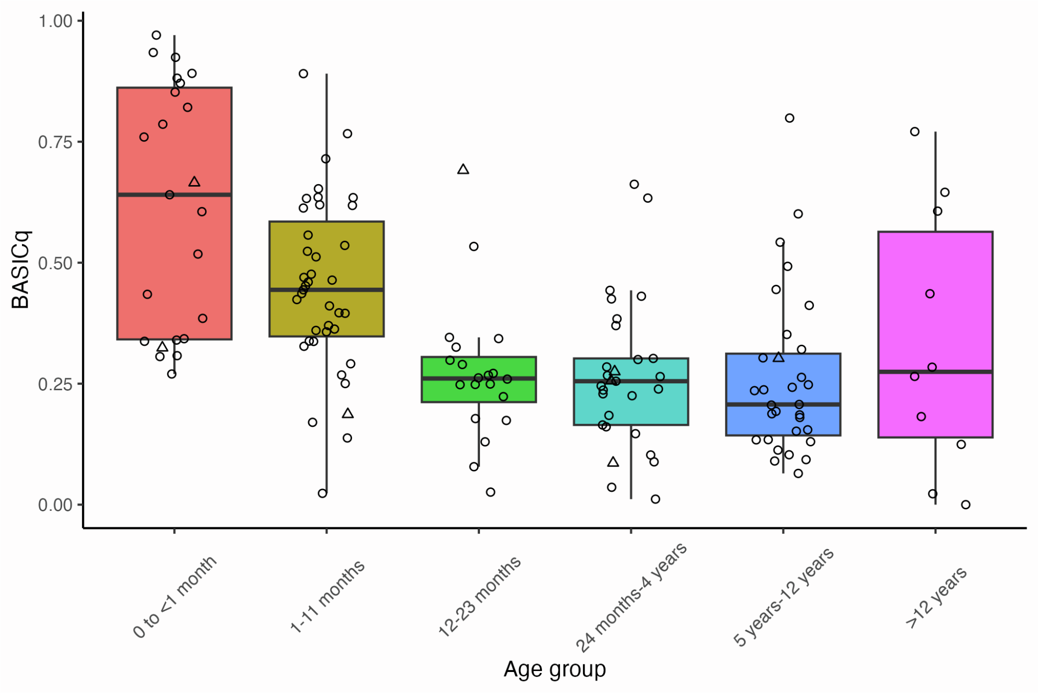

Supplemental Figure 4. BASICq score by Phoenix Sepsis Score (PSS). PSS at admission to PICU (patients with infection and PSS ≥2 only).

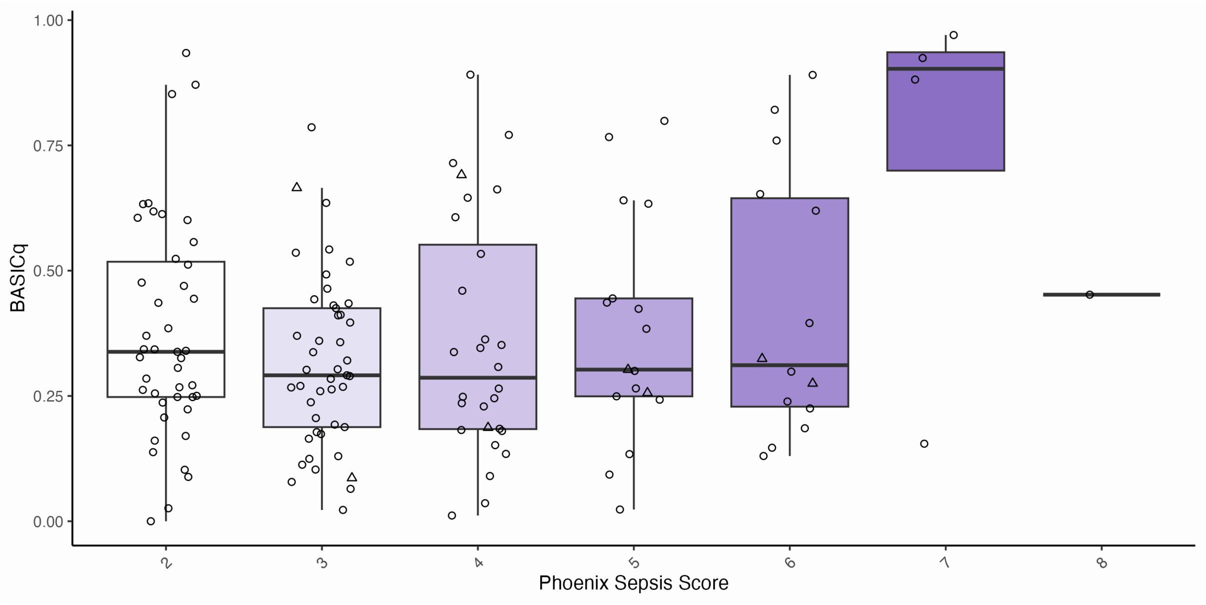

Supplemental Figure 5. Use of Cibersort to impute immune cell proportions in data. (A) Neutrophil proportion (of total white cell count) on admission to PICU (clinical laboratory) and neutrophil proportion imputed within time point 1 sample (Cibersort; unadjusted linear regression line and 95% CIs shown). (B) Lymphocyte proportion (of total white cell count) on admission to PICU (clinical laboratory) and sum of lymphocyte population proportions imputed on time point 1 sample (Cibersort; unadjusted linear regression line and 95% CIs shown). (C) Correlation (Pearson) between immune cell populations in each sample by BASIC Endotypes. (D) Imputed neutrophil proportion (Cibersort) within time point 1 sample and BASICq score (unadjusted linear regression line and 95% CIs shown). (E) Imputed immune cell populations (Cibersort) within time point 1 sample by BASIC Endotype.

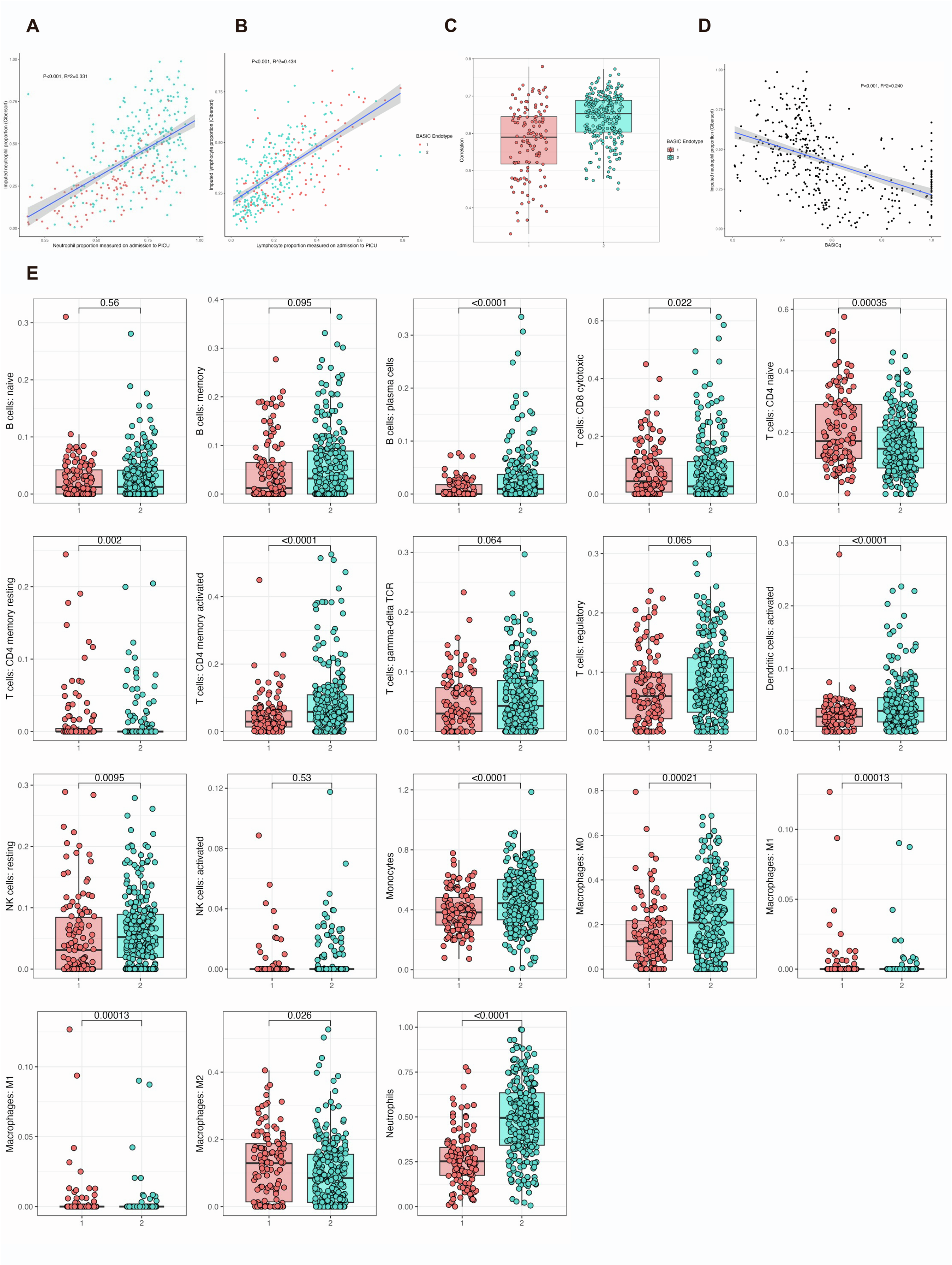
A
P<0.001, R^2=0.331
Imputed neutrophil proportion (Cibersort)
Neutrophil proportion measured on admission to PICU
B
P<0.001, R^2=0.434
Imputed lymphocyte proportion (Cibersort)
Lymphocyte proportion measured on admission to PICU
BASIC Endotype
C
Correlation
BASIC Endotype
D
P<0.001, R^2=0.240
Imputed neutrophil proportion (Cibersort)
BASICq
E
B cells: naive 0.56
B cells: memory 0.095
B cells: plasma cells <0.0001
T cells: CD8 cytotoxic 0.022
T cells: CD4 naive 0.00035
T cells: CD4 memory resting 0.002
T cells: CD4 memory activated <0.0001
T cells: gamma-delta TCR 0.064
T cells: regulatory 0.065
Dendritic cells: activated <0.0001
NK cells: resting 0.0095
NK cells: activated 0.53
Monocytes <0.0001
Macrophages: M0 0.00021
Macrophages: M1 0.00013
Macrophages: M1 0.00013
Macrophages: M2 0.026
Neutrophils <0.0001

Supplemental Figure 6. Development of a limited gene signature set and comparison with sepsis response syndromes in adults with sepsis. (A) Distribution of top 10 differentially expressed genes to discriminate between BASIC endotype 1 and BASIC endotype 2. (B) Bubble diagram describing the optimal number of differentially expressed genes (size of bubble) to achieve a threshold sensitivity and specificity of 80%. (C) Allocation of SRS membership of BASIC samples using the 7-gene "Davenport" SRS signature (*SepstratifieR*) in principal components (PC) 1 and 2. (D) Allocation of SRS membership of BASIC samples using the 19-gene "Extended" SRS signature. Distribution of SRSq and BASICq (unadjusted linear regression line and 95% CIs shown).

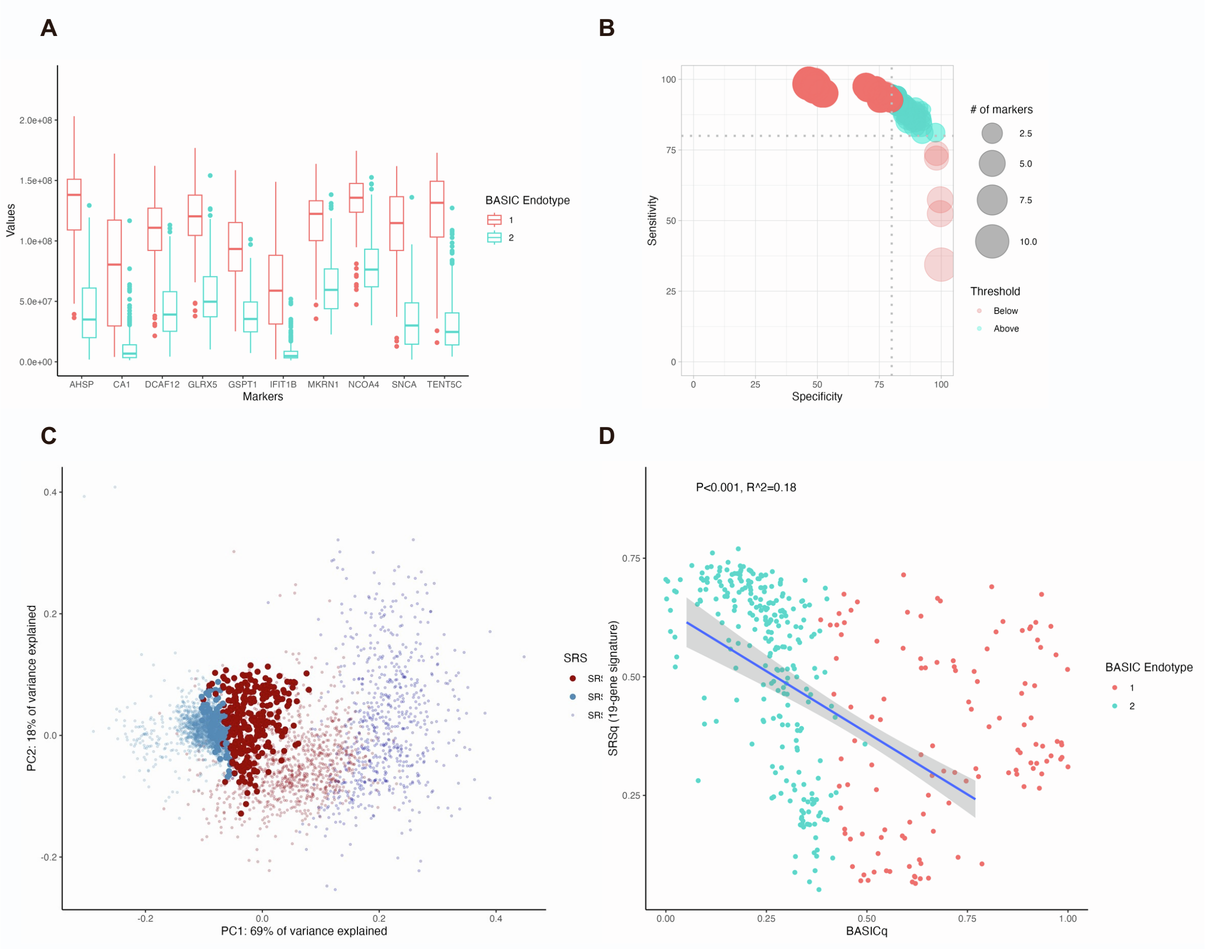
A
B
C
D
Values
Markers
AHSP
CA1
DCAF12
GLRX5
GSPT1
IFIT1B
MKRN1
NCOA4
SNCA
TENT5C
BASIC Endotype
Sensitivity
Specificity
# of markers
Threshold
Below
Above
PC2: 18% of variance explained
PC1: 69% of variance explained
SRS
P<0.001, R^2=0.18
SRSq (19-gene signature)
BASICq

SUPPLEMENTAL TABLES

Supplemental Table 1. Children on whom infection was suspected or confirmed and with a Phoenix Sepsis Score ≥2 (established sepsis).

Clinical characteristic	Patients with sepsis	BASIC endotype 1 with sepsis	BASIC endotype 2 with sepsis	p value
n	152	54 (35.5%)	98 (64.5%)	–
Sex at birth (%)				
Female	76 (50%)	25 (46.2%)	51 (52.0%)	0.611
Male	76 (50%)	29 (53.7%)	47 (48.0%)	–
Patient age group (%)				
0 to <1 month	23 (15.1%)	16 (29.6%)	7 (7.1%)	<0.001
1 to 11 months	39 (25.6%)	22 (40.7%(	17 (17.3%)	0.003
12 to 23 months	20 (13.2%)	2 (3.7%)	18 (18.4%)	0.021
24 months to 4 years	29 (19.1%)	5 (9.3%)	24 (24.5%)	0.038
5 years to 12 years	31 (20.4%)	5 (9.3%)	26 (26.5%)	0.020
>12 years	10 (6.6%)	4 (7.4%)	6 (6.1%)	1
Presence of 1 or more comorbidity (%)	76 (50%)	28 (51.9%)	48 (49.0%)	0.865
Infection status (%)				
Non-infectious (following review of all results)	24 (15.8%)	13 (24.1%)	11 (11.2%)	0.065
Infection type unknown	37 (24.3%)	14 (25.9%)	23 (23.5%)	0.888
Viral infection	40 (26.3%)	15 (27.8%)	25 (25.5%)	0.911
Bacterial infection	51 (33.6%)	12 (22.2%)	39 (39.8%)	0.044
Phoenix Sepsis Score (median, IQR, max)*				
Respiratory	2.0 (1.0–3.0, 3.0)	2.0 (1.0–3.0)	2.0 (2.0–2.8)	<0.001
Cardiovascular	1.0 (0–2.0, 6.0)	1.5 (1.0–2.0)	1.0 (0–2.0)	0.002
Coagulation**	0 (0–0, 1)	0 (0–0)	0 (0–0)	<0.001
Neurological	1.0 (0–1.0, 3.0)	1.0 (0–1.0)	1.0 (0–1.0)	<0.001
Total	3.0 (2.0–4.0, 8.0)	3.0 (2.0–5.0)	3.0 (2.0–4.0)	<0.001
*Paediatric ARDS status****				
Missing pARDS status	15 (9.9%)	4 (7.4%)	11 (11.2%)	0.398
No pARDS	43 (28.3%)	16 (29.6%)	27 (27.6%)	0.457
Mild/moderate pARDS	69 (45.4%)	22 (40.7%)	47 (48.0%)	0.854
Severe pARDS	25 (16.4%)	12 (22.2%)	13 (13.3%)	0.006
Phoenix sepsis score (median, IQR, max)	3 (2–4), 8	3 (2–5), 8	3 (2–4), 7	<0.001
PIM2 score				
Investigations				
CRP at transfer (median, IQR, max)	48 (18–111, 520)	33 (10–65, 304)	61 (31–164, 520)	<0.001
Maximum CRP (median, IQR, max)	76 (38–183, 520)	54 (34–135, 304)	88 (45–212, 520)	<0.001
Neutrophil count at transfer (median, IQR, max)	6.8 (3.5–12.3, 38.7)	4.2 (2.3–7.5, 18.3)	8.0 (5.3–15.3, 38.7)	<0.001
Treatment				
Required invasive ventilation (%)	150 (98.7%)	53 (98.1%)	97 (99.0%)	1
Required vasoactive support (%)	114 (75.0%)	44 (81.5%)	70 (71.4%)	0.240
Outcomes				
Duration of ventilation (days, median, IQR, max)	6.0 (4.0–9.3, 139)	6.0 (4.0–10.0, 139)	5.5 (3.0–8.8, 22.0)	<0.001
VFD-30 (median, IQR)	24.0 (20.8–26.0)	23.0 (16.3–25.0)	24.0 (21.0–27.0)	<0.001
Duration of ventilation ≥30 days (%)	9 (5.9%)	4 (7.4%)	5 (5.1%)	0.828

Mortality (%)	8 (5.3%)	2 (3.7%)	6 (6.1%)	0.795
Ventilation ≥30 days or mortality (%)	14 (9.2%)	6 (11.1%)	8 (8.2%)	0.758

Supplemental Table 2A. Ventilator-free days at day 30 (VFD-30) by BASIC endotype. Multiple linear regression adjusted for age, reason for PICU admission, co-morbidity, infection status and pediatric acute respiratory distress syndrome (ARDS) category[1].

Outcome	Characteristic	Estimate	95% CI	p value
VFD-30	*BASIC endotype 1 membership*	-4.447	-7.366– -1.528	**0.003**
	Sex			
	Female	–	–	–
	Male	-1.138	-3.188– 0.913	0.275
	Age			
	0 to <1 month	–	–	–
	1-11 months	-0.303	-3.603–2.996	0.856
	12-23 months	0.932	-3.577–5.442	0.684
	24 months-4 years	-2.914	-7.116–1.289	0.173
	5 years-12 years	-0.403	-4.517–3.711	0.847
	>12 years	-2.04	-7.719–3.639	0.48
	Reason for PICU admission			
	Infection/Sepsis	–	–	–
	Cardiac	-3.431	-7.870–1.008	0.129
	Endocrine/Metabolic	0.792	-14.59–16.173	0.919
	Respiratory/Airway	-0.082	-3.160–2.996	0.958
	Neurological	-0.39	-4.245–3.465	0.842
	Trauma/Head Injury	-0.845	-7.451–5.761	0.801
	Other	-2.548	-8.242–3.145	0.378
	Co-morbidity			
	None	–	–	–
	Cardiac	2.553	-1.898–7.005	0.259
	Metabolic/endocrine	-8.504	'-13.89– -3.118	**0.002**
	Respiratory	-1.551	-5.004–1.902	0.377
	Neurological	1.705	-2.676–6.085	0.444
	Haematology/Oncology	-4.632	-12.56–3.293	0.25
	Genetic syndrome	-5.415	-11.43–0.604	0.078
	Multi-system disorder	5.799	-9.575–21.17	0.458
	Other	-1.211	-4.203–1.780	0.426
	Infection status			
	Non-infectious	–	–	–
	Infection type unknown	-0.362	-4.123–3.400	0.85
	Viral	-0.062	-3.640–3.516	0.973
	Bacterial	-1.598	-5.455–2.259	0.415
	pARDS category			
	None	–	–	–
	Mild/moderate	-2.28	-4.586–0.026	0.053
	Severe	-2.962	-6.089–0.166	0.063

Supplemental Table 2B. Ventilator-free days at day 30 (VFD-30) by BASIC endotype membership. Multiple linear regression adjusted for age, reason for PICU admission, co-morbidity, infection status and Phoenix Sepsis Score (PSS) in all patients with infection and PSS ≥2 (i.e. a diagnosis of sepsis).

Outcome	Characteristic	Estimate	95% CI	p value
VFD-30	*BASIC endotype 1 membership*	-2.757	-5.479– -0.035	**0.049**
	Sex			
	Female	–	–	–
	Male	-1.941	-4.298– 0.416	0.109
	Age			
	0 to <1 month	–	–	–
	1-11 months	1.587	-2.163– 5.338	0.408
	12-23 months	2.635	-2.091–7.361	0.277
	24 months-4 years	-1.474	-5.900–2.953	0.515
	5 years-12 years	0.679	-3.834–5.193	0.768
	>12 years	2.49	-3.367–8.347	0.406
	Reason for PICU admission			
	Infection/Sepsis	–	–	–
	Cardiac	-3.018	-9.160–3.124	0.337
	Endocrine/Metabolic	5.628	-8.870–20.13	0.448
	Respiratory/Airway	0.418	-3.338–4.173	0.828
	Neurological	6.327	0.123–12.53	**0.048**
	Trauma/Head Injury	5.562	-5.051–16.17	0.306
	Other	6.557	0.670–12.44	**0.031**
	Co-morbidity			
	None	–	–	–
	Cardiac	-2.495	-10.80–5.814	0.557
	Metabolic/endocrine	-7.791	-13.07– -2.509	**0.005**
	Respiratory	-2.404	-6.130–1.323	0.208
	Neurological	-4.928	-10.63–0.775	0.093
	Haematology/Oncology	-2.25	-13.23–8.735	0.689
	Genetic syndrome	-3.926	-8.543–0.690	0.098
	Multi-system disorder	7.23	-6.918–21.38	0.318
	Other	-0.593	-4.152–2.966	0.745
	Infection status			
	Non-infectious	–	–	–
	Infection type unknown	2.107	-2.525–6.739	0.374
	Viral	0.546	-3.903–4.994	0.81
	Bacterial	1.596	-3.159–6.352	0.512
	PSS (score 2–9)	-0.938	-1.757– -0.119	**0.026**

Supplemental Table 3. Comparison of BASICq score by age group.

Age group	BASICq (median, IQR)	p value for comparison of BASICq between age groups					
		0 to <1 month	1–11 months	12–23 months	24 months –4 years	5 years–12 years	>12 years
0 to <1 month	0.77 (0.52–0.92)	–	<0.001	<0.001	<0.001	<0.001	<0.001
1–11 months	0.41 (0.32–0.53)	<0.001	–	<0.001	<0.001	<0.001	<0.001
12–23 months	0.27 (0.22–0.34)	<0.001	<0.001	–	0.892	0.132	0.865
24 months –4 years	0.27 (0.22–0.34)	<0.001	<0.001	0.892	–	0.048	0.839
5 years–12 years	0.23 (0.16–0.32)	<0.001	<0.001	0.132	0.048	–	0.200
>12 years	0.27 (0.21–0.33)	<0.001	<0.001	0.865	0.839	0.200	–

Supplemental Table 4. Comparison of BASICq score by infection status.

	BASICq (median, IQR)	p value for comparison of BASICq between reasons for admission			
		Non-infectious	Unknown	Viral	Bacterial
Non-infectious	0.35 (0.26–0.76)	–	0.005	0.059	<0.001
Infection unknown	0.30 (0.21–0.43)	0.005	–	0.147	0.024
Viral	0.34 (0.27–0.44)	0.059	0.147	–	<0.001
Bacterial	0.24 (0.15–0.37)	<0.001	0.024	<0.001	–

Supplemental Table 5. Comparison of BASICq score by reason for admission.

Reason for admission	BASICq (median, IQR)	p value for comparison of BASICq between reasons for admission						
		Infection/Sepsis	Cardiac	Endocrine/Metabolic	Respiratory/Airway	Neurological	Trauma/Head Injury	Other
Infection/Sepsis	0.31 (0.21–0.42)	–	<0.001	0.295	0.072	0.705	0.075	0.112
Cardiac	0.72 (0.58–0.91)	<0.001	–	0.004	<0.001	<0.001	<0.001	<0.001
Endocrine/Metabolic	0.34 (0.31–0.40)	0.295	0.004	–	0.629	0.060	0.059	0.477
Respiratory/Airway	0.31 (0.21–0.64)	0.072	<0.001	0.629	–	0.256	0.026	0.024
Neurological	0.29 (0.26–0.34)	0.705	<0.001	0.060	0.256	–	–	0.004
Trauma/Head Injury	0.24 (0.19–0.33)	0.075	<0.001	0.059	0.026	0.138	–	<0.001
Other	0.46 (0.28–0.56)	0.112	<0.001	0.477	0.024	0.004	<0.001	–

Supplemental Table 6. Comparison of BASICq score by age group in patients with infection and PSS ≥2 (i.e. a diagnosis of sepsis).

Age group	BASICq (median, IQR)	p value for comparison of BASICq between age groups					
		0 to <1 month	1–11 months	12–23 months	24 months –4 years	5 years–12 years	>12 years
0 to <1 month	0.64 (0.34–0.86)	–	0.030	<0.001	<0.001	<0.001	0.007
1–11 months	0.44 (0.35–0.59)	0.030	–	<0.001	<0.001	<0.001	0.142
12–23 months	0.21 (0.26–0.31)	<0.001	<0.001	–	0.770	0.383	0.779
24 months –4 years	0.26 (0.16–0.30)	<0.001	<0.001	0.770	–	0.617	0.646
5 years–12 years	0.21 (0.14–0.31)	<0.001	<0.001	0.383	0.617	–	0.580
>12 years	0.27 (0.14–0.56)	0.007	0.142	0.779	0.646	0.580	–

Supplemental Table 7. Comparison of BASICq score by Phoenix Sepsis Score (PSS) in patients with infection and PSS ≥2 (i.e. a diagnosis of sepsis).

Phoenix Sepsis Score	BASICq (median, IQR)	p value for comparison of BASICq between PSS						
		2	3	4	5	6	7	8
2	0.34 (0.25–0.52)	_	0.227	0.571	0.987	0.790	0.083	0.682
3	0.29 (0.19–0.43)	0.227	–	0.765	0.443	0.301	0.053	0.391
4	0.29 (0.18–0.55)	0.571	0.765	–	0.635	0.501	0.072	0.690
5	0.30 (0.25–0.44)	0.987	0.443	0.635	–	0.860	0.080	0.556
6	0.31 (0.23–0.64)	0.790	0.301	0.501	0.860	–	0.127	0.800
7	0.90 (0.70–0.94)	0.083	0.053	0.072	0.080	0.127	–	0.800
8	0.45	0.682	0.391	0.690	0.556	0.800	0.800	–

SUPPLEMENTAL REFERENCES

1. Emeriaud, G., et al., *Executive Summary of the Second International Guidelines for the Diagnosis and Management of Pediatric Acute Respiratory Distress Syndrome (PALICC-2).* Pediatr Crit Care Med, 2023. **24**(2): p. 143-168.
